# Evaluation of silver nanoparticles for controlling *Lipaphis erysimi* and *Alternaria brassicicola* and assessing their effects on Chinese kale growth and soil properties

**DOI:** 10.3389/finsc.2026.1911760

**Published:** 2026-09-11

**Authors:** Sarayut Pittarate, Perumal Vivekanandhan, Kannan Swathy, Thanandon Siripan, Sukanya Haituk, Rapeephun Dangtungee, Patcharin Krutmuang

**Affiliations:** 1 Office of Research Administration, Chiang Mai University, Chiang Mai, Thailand; 2 Department of Entomology and Plant Pathology, Faculty of Agriculture, Chiang Mai University, Chiang Mai, Thailand; 3 Center of Excellence on Agricultural Biotechnology, Office of the Permanent Secretary, Ministry of Higher Education, Science, Research, and Innovation (AG-BIO/MHESI), Bangkok, Thailand; 4 Center of Omics for High-Value Agriculture, Chiang Mai University (AgOmics-CMU), Faculty of Agriculture, Chiang Mai University, Chiang Mai, Thailand; 5 International Industry and Agriculture Innovative Research Center, Maejo University International College, Chiang Mai, Thailand

**Keywords:** *Alternaria brassicicola*, Chinese kale (*Brassica oleracea* var. alboglabra), chlorophyll content, insecticidal activities, plant physiological traits, silver nanoparticles (AgNPs), soil nutrients

## Abstract

**Introduction:**

Chinese kale (*Brassica oleracea* var. alboglabra) is an important leafy vegetable that is highly susceptible to the mustard aphid, *Lipaphis erysimi*, and the fungal pathogen *Alternaria brassicicola*, which can reduce crop yield and quality. This study evaluated the efficacy of silver nanoparticles (AgNPs) against *L. erysimi* and *A. brassicicola* and assessed their effects on plant physiological characteristics and soil nutrient status.

**Methods:**

AgNPs at different concentrations were evaluated for their toxicity against *L. erysimi* and their inhibitory effects on the mycelial growth and spore germination of *A. brassicicola*. The effects of AgNPs on Chinese kale growth, chlorophyll content, and selected soil chemical properties were also assessed.

**Results:**

AgNPs exhibited significant dose- and time-dependent toxicity against *L. erysimi*, with LC₅₀ values decreasing from 43.109 ppm at 24 h to 19.343 ppm at 48 h. Aphid populations were reduced by 88.33% following treatment with 100 ppm AgNPs for 48 h. AgNPs at 80–100 ppm also significantly inhibited the mycelial growth and spore germination of *A. brassicicola*. However, higher AgNP concentrations reduced chlorophyll content and adversely affected fresh weight and leaf development. Soil analysis showed slight increases in pH and organic matter, whereas available phosphorus and exchangeable potassium decreased following AgNP application.

**Discussion:**

These findings indicate that AgNPs have potential as a component of integrated pest and disease management for Chinese kale. However, optimization of application rates is necessary to balance pest and disease suppression with potential adverse effects on plant growth and soil properties. Further studies under field conditions are needed to determine appropriate application rates and evaluate their long-term environmental effects.

## Introduction

Chinese kale (*Brassica oleracea* var. alboglabra) is a leafy vegetable that is grown a lot in Southeast Asia and is important to the economy. It is high in nutrients and can be used in many different ways in cooking (1, 2). However, insect pests and fungal diseases make it very hard to grow, which can greatly lower both the amount and quality of the crop (1, 2). The mustard aphid, *Lipaphis erysimi* (Kalt.), is one of the most worrisome pests because it feeds on phloem directly and spreads viral pathogens indirectly (3–5). Fungal pathogens like *Alternaria brassicicola* are also common in Brassica crops (6). They can cause leaf spots, blight, and lower the marketability of the crops (6, 7). Conventional chemical pesticides have been employed to mitigate these biotic stresses; however, their excessive application has elicited apprehensions regarding environmental contamination, pesticide resistance, and residue accumulation in food products (8–11).

Recent advancements in nanotechnology have facilitated the creation of sustainable and environmentally friendly approaches for pest and disease management (12–17). Silver nanoparticles (AgNPs) are one type of nanomaterial that has gotten a lot of attention because they are very effective at killing bacteria, insects, and larvae, are very small, and have a high surface-to-volume ratio. These traits work together to make them more effective biologically, even in small amounts (12, 13, 18–21). Many studies have shown that AgNPs work by breaking down cell membranes, making reactive oxygen species, and messing with important metabolic pathways in insects and harmful microorganisms. They also pose less risk to the environment than traditional synthetic agrochemicals (9, 22–27).

Green synthesis methods that use plant and marine resources have made it even easier to make nanoparticles that are safe for living things and last a long time (28, 29). For instance, the environmentally friendly synthesis of silver nanoparticles using the seagrass *Cymodocea serrulata* (R. Br.) Asch. & Magnus showed significant anticancer, antioxidant, and antiglycemic effects (30). In the same way, the eco-friendly phytofabrication of AgNPs using the aqueous extract of *Aristolochia bracteolata* Lam showed strong antioxidant potential, antibacterial activity against clinical pathogens, and malarial larvicidal efficacy in the lab (31).

Other metal nanoparticles made in a green way, in addition to silver-based ones, have also shown promise as pesticides. A green copper-based nano-pesticide made from *Annona squamosa* L. seeds was said to work well against insect pests and not hurt other organisms (10). Moreover, zinc oxide nanoparticles biosynthesized from the marine alga *Sargassum ilicifolium* and *Annona squamosa* L. seeds extract-derived AgNPs have demonstrated larvicidal properties and enhanced tomato plant resistance against *Tuta absoluta* (32, 33), as well as alleviating the detrimental effects of whitefly infestation by augmenting plant defense mechanisms (34). Limited research has assessed the impact of AgNPs on pest and pathogen management, plant physiological responses, and soil nutrient levels in Chinese kale, despite their potential. The current study seeks to evaluate the impact of silver nanoparticles (AgNPs) on the population dynamics of *L. erysimi*, the growth and spore germination of *A. brassicicola*, the physiological and biochemical parameters of Chinese kale, and the nutrient status of the soil under semi-field conditions. This research offers a thorough assessment of AgNPs as an environmentally friendly and sustainable method for integrated pest and disease management in the cultivation of Chinese kale by examining various facets.

## Materials and methods

### Source of silver nanoparticles

Extra-pure silver nanoparticles (AgNPs; 20,000 ppm) were obtained from NAVATECH Co., Ltd. (Chiang Mai, Thailand). The private company precursor used to synthesize colloidal silver was 99.99% of silver metal component dissolved in nitric acid (HNO_3_) (35). Ethylene glycol (EG) was used as a solvent for the AgNO_3_ and a reducing agent. Polyvinylpyrrolidone (PVP) was used as a polymeric capping agent. The transparent solution first obtained a characteristic pale pink and then a gray-green color, which indicated the formation of silver nanoparticles (35). The final concentration of the product was 20,000 ppm.

Although the AgNPs were supplied as stable aqueous suspensions, aggregation can occur upon storage; thus, all nanoparticle suspensions were vortex-mixed for 10–20 min immediately prior to use to ensure proper dispersion.

### Preparation of AgNPs working suspensions

The AgNPs stock suspension was stabilized with polyvinyl-pyrrolidone (PVP). Before each bioassay, the stock suspension was vortex-mixed for 60 s to redisperse any particles that may have settled during storage. The required working concentrations of 20, 40, 60, 80, and 100 ppm were prepared by dilution with deionized water. Each freshly diluted working suspension was vortex-mixed for an additional 30 s and applied within 5 min after preparation. Vortex mixing was used solely as an immediate redispersion procedure and was not considered evidence of long-term colloidal stability or permanent uniformity of the nanoparticle suspension.

### The effects of AgNPs on the population size of *L. erysimi* on Chinese kale plants

#### Propagation of Chinese kale

Chinese kale seedlings at the 1–2 true leaf stage were transplanted from seed trays into plastic pots (10 cm in diameter and 15 cm in depth) filled with unsterilized compost. The plants were grown for three weeks in nylon cages (50 cm × 50 cm × 50 cm) under controlled conditions of 25 ± 5 °C and a 12:12 h light:dark photoperiod. Regular irrigation was provided throughout the experimental period, and fifteen plants were assigned to each treatment.

### Insect rearing and maintenance

The mustard aphid (*Lipaphis erysimi*) colony used in this study was obtained from a stock culture maintained at the Division of Entomology, Department of Entomology and Plant Pathology, Faculty of Agriculture, Chiang Mai University, Thailand. The stock culture was reared on healthy Chinese kale (*Brassica alboglabra* L.H. Bailey) plants grown in pots and maintained in nylon cages (50 × 50 × 50 cm) under controlled conditions of 25 ± 5 °C and a 12:12 h light photoperiod (36). Second- and third-instar nymphs from this colony were used for the bioassays, while apterous adult females (2–3 days after the final molt) were selected for the population growth experiments. Aphids were carefully transferred using a fine camel-hair brush and gently placed onto the leaves of Chinese kale plants.

Ethical approval for this study was obtained from the Animal Care and Use Committee, Faculty of Agriculture, Chiang Mai University, Thailand (Protocol No. AG04001/2568). All procedures involving *L. erysimi* were conducted in accordance with national ethical guidelines and institutional standard operating procedures (SOPs)."

### Effect of insecticidal activities of AgNPs on *L. erysimi*

The tested silver nanoparticles (AgNPs) were dispersed in sterile distilled water to prepare a 500ppm stock suspension. Five concentrations (20, 40, 60, 80, and 100ppm) were prepared for the insecticidal activities bioassay against *L. erysimi*. Sterile distilled water without AgNPs served as the control. For each replicate, twenty second to third instar nymphs were transferred onto excised Chinese kale leaf discs (45 mm diameter) placed in 45 mm plastic Petri dishes containing 0.3% water agar to maintain leaf turgidity. AgNPs suspensions were applied using a calibrated 5 mL hand held micro sprayer, and 1 mL of suspension was sprayed evenly onto the aphids and both surfaces of the leaf disc until complete coverage was achieved without excessive runoff. Following treatment, the Petri dishes were immediately covered with lids and maintained under controlled laboratory conditions (25 ± 2 °C, 65 ± 5% relative humidity, and a 12:12h light:dark photoperiod) (37). Aphid mortality was assessed under a stereomicroscope at 24 and 48 h after treatment. Aphids were considered dead when they exhibited no movement after gentle stimulation with a fine camel-hair brush. Each treatment consisted of three biological replicates.

### Effect of AgNPs on *L. erysimi* population size

Six-week-old Chinese kale plants were individually placed in nylon mesh cages (50 cm in diameter × 50 cm in height), and ten adult *L. erysimi* were introduced onto the middle leaf of each plant following the method described in Ref. (38). AgNP suspensions at the designated concentrations were applied using a 30-mL handheld microsprayer. Each plant received 10 mL of the respective suspension, which was uniformly sprayed over the foliage to ensure complete coverage. The treatments were applied once daily for seven consecutive days following the procedure described in Ref. (38). Control plants were sprayed with an equal volume of sterile distilled water.

The cages were securely enclosed with fine no-see-um mesh to prevent aphid escape and cross-contamination among treatments. The experiment consisted of six treatments, with three replicates per treatment and five plants per replicate, resulting in a total of 90 caged plants per experimental run. The plants were randomly arranged and maintained under natural environmental conditions (25–30 °C and 30–80% relative humidity). The total aphid population, including both adults and nymphs, on each plant was recorded 24 h after the final application using a hand magnifier. The entire experiment was independently conducted twice to confirm the reproducibility of the results."

### The effects of AgNPs on physiological and biochemical parameters of Chinese kale

All six-week-old Chinese kale plants (n = 45 per treatment) were examined, and the number of fully developed leaves and stems was recorded for each plant.

### Analysis of chlorophyll content

To evaluate the effect of silver nanoparticles (AgNPs) on total chlorophyll content (chlorophyll a and b) in seven-week-old Chinese kale seedlings, three independent biological replicates were analyzed for each treatment. For each replicate, approximately 100 g of fresh leaves were randomly collected, chopped, and homogenized separately. Subsequently, 1 g of the homogenized leaf tissue from each replicate was transferred to a 50-mL beaker and extracted with 25 mL of 80% (v/v) acetone. The mixture was allowed to stand for 1 h to ensure adequate pigment extraction (38).

The homogenate was then filtered through Whatman No. 1 filter paper, and the absorbance of the filtrate was measured using a spectrophotometer (Thermospectronic). Eighty percent acetone was used as a blank to zero the instrument before measurement and between wavelength changes. Chlorophyll a and chlorophyll b absorbance values were recorded at 663 nm and 645 nm, respectively, following the method described by (38).

Chlorophyll concentrations were calculated on a fresh weight basis, and each sample was analyzed in triplicate. The chlorophyll content was determined using the following equations:


$$\begin{array}{l} chlorophyl\;a(mg\;g{-}^{1})=[12.7(OD663)-2.69(OD645)]\times V/(1000\;*\;W)\\ chlorophyl\;b(mg\;g{-}^{1})=[22.9(OD645)-4.68(OD663)]\times V/(1000\;*\;W)\\ total\;chlorophyl\;content(mg\;g{-}^{1})=[20.2(OD645)+8.02(OD663)]\times V/(1000\;*\;W) \end{array}$$

where OD₆₆₃ and OD₆₄₅ represent the optical density (absorbance) values measured at 663 and 645 nm, respectively; V is the final volume of the chlorophyll extract (mL); and W is the fresh weight of the plant tissue used for extraction (g). The coefficients in the equations are specific absorption coefficients used to calculate chlorophyll a, chlorophyll b, and total chlorophyll concentrations while accounting for the overlapping absorption spectra of chlorophyll a and b.

### The effects of AgNPs on Chinese kale fungus pathogens

#### Source of fungal inoculum

In this study, the fungal isolate *Alternaria brassicicola* CDEP-239 was obtained from the pure culture collection of the Department of Entomology and Plant Pathology (CDEP), Faculty of Agriculture, Chiang Mai University, Chiang Mai, Thailand. The isolate was characterized and taxonomically identified following the protocols described by (39).

### Effect of AgNPs on mycelial inhibition

The antifungal activity of silver nanoparticles (AgNPs) against *A. brassicicola* isolate CDEP-239 was evaluated using the poisoned food technique, following the method described by (37). AgNPs were tested at concentrations of 20, 40, 60, 80 and 100 ppm, while PDA plates without AgNPs served as negative controls. Potato Dextrose Agar (PDA) medium was amended with the respective concentrations of AgNPs prior to solidification, and agar plugs (5 mm diameter) taken from the actively growing margins of the fungal culture were placed at the center of each Petri dish. All treatments were conducted in triplicate, and mean values were calculated. Radial (linear) mycelial growth was measured when the control plates reached full growth, as described by (40). The percentage inhibition of mycelial growth for each treatment was calculated using the standard formula.


Inhibition percentage (I) = (C-T)/C×100

Where:

I= Percent inhibition,

C = Colony diameter in control (mm),

T = Colony diameter in respective treatment (mm).

The data on percent inhibition were transformed in arcs and statistically examined using a completely randomized design (CRD).

### Effect of AgNPs on spore germination

The inhibitory effect of silver nanoparticles (AgNPs) on conidial germination of *A. brassicicola* isolate CDEP-239 was evaluated with minor modifications to the method described by (39). A 100 μL aliquot of conidial suspension (1 × 10^6^ conidia mL^-^¹) was mixed with an equal volume (100 μL) of AgNP suspension to obtain final AgNP concentrations of 20, 40, 60, 80, and 100 ppm. Sterile distilled water was used instead of AgNPs in the control treatment. Each mixture was evenly spread onto Potato Dextrose Agar (PDA) plates and incubated at 25 ± 2 °C under dark conditions. Conidial germination was evaluated at 3, 6, 9, 12 and 24 h after inoculation. At each observation time, 100 randomly selected conidia were examined under a Leica, Thunder 3D tissue and DM6B, (HISTOCENTER, Bangkok, Thailand) compound microscope (400× magnification) for each replicate, and conidia were considered germinated when the germ tube length was equal to or greater than the diameter of the conidium. Three independent replicates were performed for each treatment. Results were expressed as the percentage of germinated conidia relative to the control. The percentage inhibition of conidial germination was calculated according to the formula described by (41):


Percentage of spore germination inhibition = (A – B)×100/A

Where:

A = average number of spores germinated in the control

B = average number of spores germinated in the test set

### Effect of AgNPs on soil

After 35 days of AgNPs application, changes in soil pH and nutrient composition were evaluated in the rhizosphere soil of Chinese kale plants. Soil analysis was conducted only for the treatment receiving 100 ppm AgNPs, which showed the highest efficacy against *L. erysimi*, and was compared with the negative control (distilled water). Rhizosphere soil was collected from the 0–10 cm depth of all pots within each treatment. The collected soil samples from each treatment were randomly pooled to form a composite sample, from which approximately 1 kg of soil was randomly subsampled for laboratory analysis. The effects of AgNPs on rhizosphere soil properties were assessed by determining soil pH and exchangeable nutrient levels. Soil pH was measured using a pH/conductometer (Metrohm; MS Scientific Instrument Co., Ltd.), and exchangeable soil nutrients were analyzed using an Atomic Absorption Spectrophotometer (Analytik Jena, ContrAA 800, Germany) following standard analytical procedures.

### Statistical analysis

Data on aphid mortality, aphid population reduction, mycelial growth inhibition, spore germination inhibition, plant growth parameters, and soil chemical properties are presented as mean ± standard error (SE). Mortality data were corrected for control mortality using Abbott’s formula prior to analysis (42). Corrected mortality data were subjected to Probit analysis to estimate the median lethal concentration (LC_50_) and ninety percent lethal concentration (LC_90_), together with their 95% confidence intervals (95% CI) and slope ± standard error (SE). All other data were analyzed by one-way analysis of variance (ANOVA), and treatment means were separated using Tukey’s honestly significant difference (LSD) test at the 5% significance level (*P*< 0.05). Statistical analyses were performed using IBM SPSS Statistics version 16.0 (IBM Corp., Armonk, NY, USA).

## Results

### Effect of insecticidal activity of AgNPs on *L. erysimi*

The insecticidal activity of silver nanoparticles (AgNPs) against *L. erysimi* was evaluated at 24 and 48 h of exposure across a range of concentrations (20–100 ppm) (**Table 1**). At 24 h, mortality in the water-treated control was 0.00 ± 0.00%, and increased progressively with increasing AgNPs concentration, from 35.00 ± 5.00% at 20 ppm to 46.66±1.66% at 40 ppm, 50.00±2.88% at 60 ppm, 63.33 ± 3.33% at 80 ppm, and reaching a maximum of 76.66 ± 2.88% at 100 ppm. At 48 h, overall mortality was higher across all treatments compared with 24 h. The control showed a low background mortality of 1.66 ± 1.66%, while mortality in the AgNPs treatments ranged from 55.00 ± 2.88% at 20 ppm to 56.66 ± 5.77% at 40 ppm, 71.66 ± 1.66% at 60 ppm, 75.00 ± 1.66% at 80 ppm, and 88.33 ± 3.33% at 100 ppm, again the highest mortality observed among all treatments. Probit analysis confirmed the concentration and time dependent toxicity of AgNPs against *L. erysimi* (**Table 1**). The LC_50_ decreased from 43.109 ppm (95% CI: 30.95–55.10) at 24 h to 19.343 ppm (95% CI: 7.38–28.42) at 48 h, while the LC_90_ decreased from 371.826 ppm (95% CI: 197.58–1760.99) at 24 h to 204.567 ppm (95% CI: 121.24–841.64) at 48 h. This roughly two-fold reduction in both LC_50_ and LC_90_ values indicates that a substantially lower concentration of AgNPs was needed to achieve equivalent mortality with longer exposure time. The slopes of the probit regression lines were 1.370 ± 0.304 (24 h) and 1.251 ± 0.311 (48 h), and the chi-square goodness of fit values (χ² = 4.919 and 3.917, respectively) indicated that the probit model adequately described the observed mortality data at both exposure times.

**Table 1:** Insecticidal activities of AgNPs against *L. erysimi*.

Exposure time (h)	Concentration (ppm)	Mortality (%)	LC_50_ (Lower-Upper)	LC_90_ (Lower-Upper)	Slope ± SE	χ²
24	Control (non)	0.00 ± 0.00	43.109 (30.95-55.10)	371.826 (197.58-1760.99)	1.370 ± 0.304	4.919
	20	35.00 ± 5.00				
	40	46.66 ± 1.66				
	60	50.00 ± 2.88				
	80	63.33 ± 3.33				
	100	76.66 ± 2.88				
48	Control (non)	1.66 ± 1.66	19.343 (7.38-28.42)	204.567 (121.24-841.64)	1.251 ± 0.311	3.917
	20	55.00 ± 2.88				
	40	56.66 ± 5.77				
	60	71.66 ± 1.66				
	80	75.00 ± 1.66				
	100	88.33 ± 3.33				

*LC_50_ values were estimated by Probit analysis using IBM SPSS Statistics. Data are presented as LC_50_ with 95% confidence intervals (95% CI) and slope ± SE.

### Effect of AgNPs on *L. erysimi* population size

**Figure 1:**
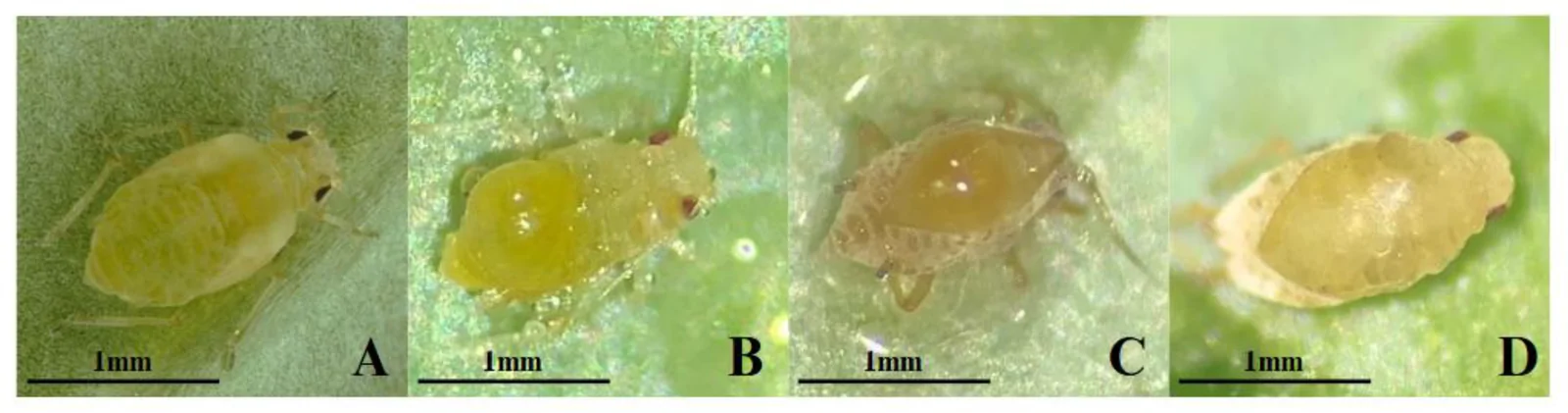
AgNPs effects on molting **(A)** control, **(B–D)** 3^rd^-4^th^ instar.

**Figure 2:**
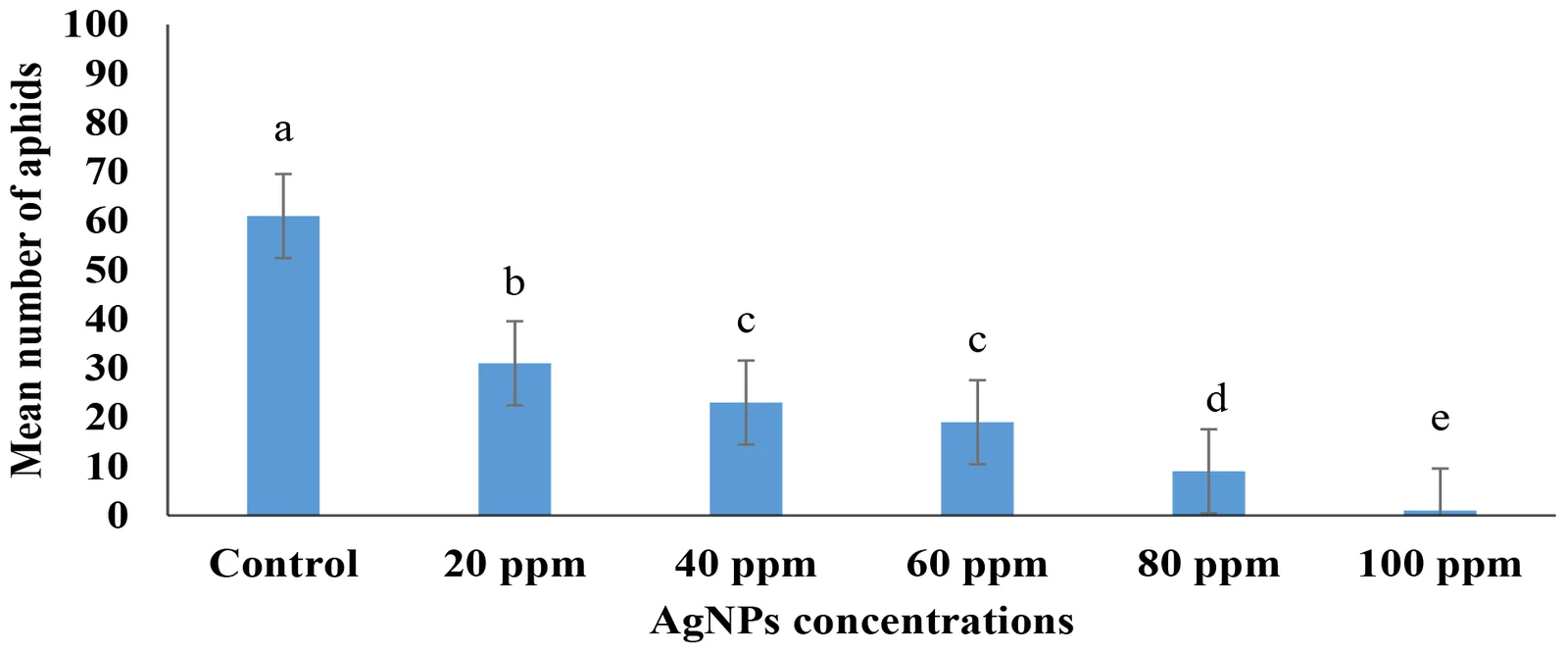
Mean (± SE) number of aphids per Chinese kale inoculated with AgNPs and distilled water (control). Bars with different letters are significantly different at *p<* 0.05 (Tukey’s Least Significant Difference (LSD), after one-way ANOVA).

The use of silver nanoparticles (AgNPs) has reduced the population of *L. erysimi* on Chinese kale compared with the control and adversely affected aphid molting, resulting in unsuccessful or incomplete molts (**Figure 1**). The average number of aphids per plant in the untreated control group was 61.66 ± 6.95. The use of AgNPs caused a decrease in the number of aphids that depended on the concentration. The population dropped to 31.93 ± 8.13 at 20 ppm. At 40 ppm and 60 ppm, it dropped even more, to 23.93 ± 4.94 and 19.13 ± 5.72, respectively. The most effective concentrations were 80 ppm and 100 ppm, which lowered aphid populations to 9.80 ± 2.24 and 1.86 ± 1.86, respectively (**Figure 2**). One-way ANOVA and Tukey’s test (*p*< 0.05) showed that all of the AgNPs treatments were very different from the control. Additionally, varying concentrations exhibited significant differences from one another, with the exception of 40 ppm and 60 ppm, which did not demonstrate significant disparity (**Figure 2**). These results show that AgNPs have a clear dose-dependent effect on reducing the number of *L. erysimi*.

### Effects of AgNPs on physiological and biochemical parameters of Chinese kale

#### Physiological parameters

The physiological effects of silver nanoparticles (AgNPs) on 7-week-old Chinese kale plants were evaluated by measuring weight, width, length and the number of leaves under different AgNPs concentrations (**Table 2**). Plant weight exhibited a decreasing trend with increasing AgNPs concentration, with the highest weight recorded in control plants (71.15 g) and the lowest at 100 ppm (33.62 g). Significant differences in weight were observed between some treatment groups (*p*< 0.001), indicating a dose-dependent inhibitory effect on plant biomass. Leaf width was also affected by AgNPs treatment, with control plants exhibiting the largest leaf width (1.29 cm), while the 100-ppm treatment showed a significant reduction (0.93 cm) compared to the control (*p* = 0.004). However, no significant differences were observed in leaf length across treatments (*p* = 0.122), with values ranging from 31.32 cm to 34.65 cm. The number of leaves varied significantly among treatments (*p* <0.001), with the 40-ppm group producing the highest number of leaves (12.26), while the 80-ppm group showed the lowest leaf count (9.33). These findings suggest that while AgNPs impact certain physiological parameters such as weight, leaf width, and leaf number in Chinese kale, leaf length remains relatively unaffected.

**Table 2:** Effects of AgNPS on physiological in 7-week-old plants grown.

Treatments (AgNPs: ppm)	Parameters			
	Weight (g)	Width (cm)	Length (cm)	Number of leaves
20	56.37 ± 3.33 bc*	1.21 ± 0.07 b	33.65 ± 2.13	11.13 ± 0.35 ab
40	46.91 ± 6.24 ab	1.19 ± 0.09 b	34.65 ± 1.77	12.26 ± 0.58 b
60	42.25 ± 2.93 ab	1.08 ± 0.06 ab	34.03 ± 1.33	10.60 ± 0.58 ab
80	42.19 ± 6.90 ab	1.15 ± 0.02 ab	32.68 ± 2.05	9.33 ± 0.23 a
100	33.62 ± 3.86 a	0.93 ± 0.04 a	31.32 ± 1.17	10.86 ± 0.55 ab
Control	71.15 ± 7.81 c	1.29 ± 0.05 a	34.51 ± 1.49	10.40 ± 0.43 ab
*p*< 0.05	< 0.001	0.004	0.122	< 0.001
*df*	5	5	5	5
SEM**	2.53	0.02	0.70	0.21

*a, b and c means different letters within the same column indicate a significant differences. ANOVA- Tukey’s Least Significant Difference (LSD) (at *p*< 0.05) was used to determine which concentrations were significantly different, **SEM, Standard error of the mean.

### Effect of AgNPs on chlorophyll content in Chinese kale

**Figure 3:**
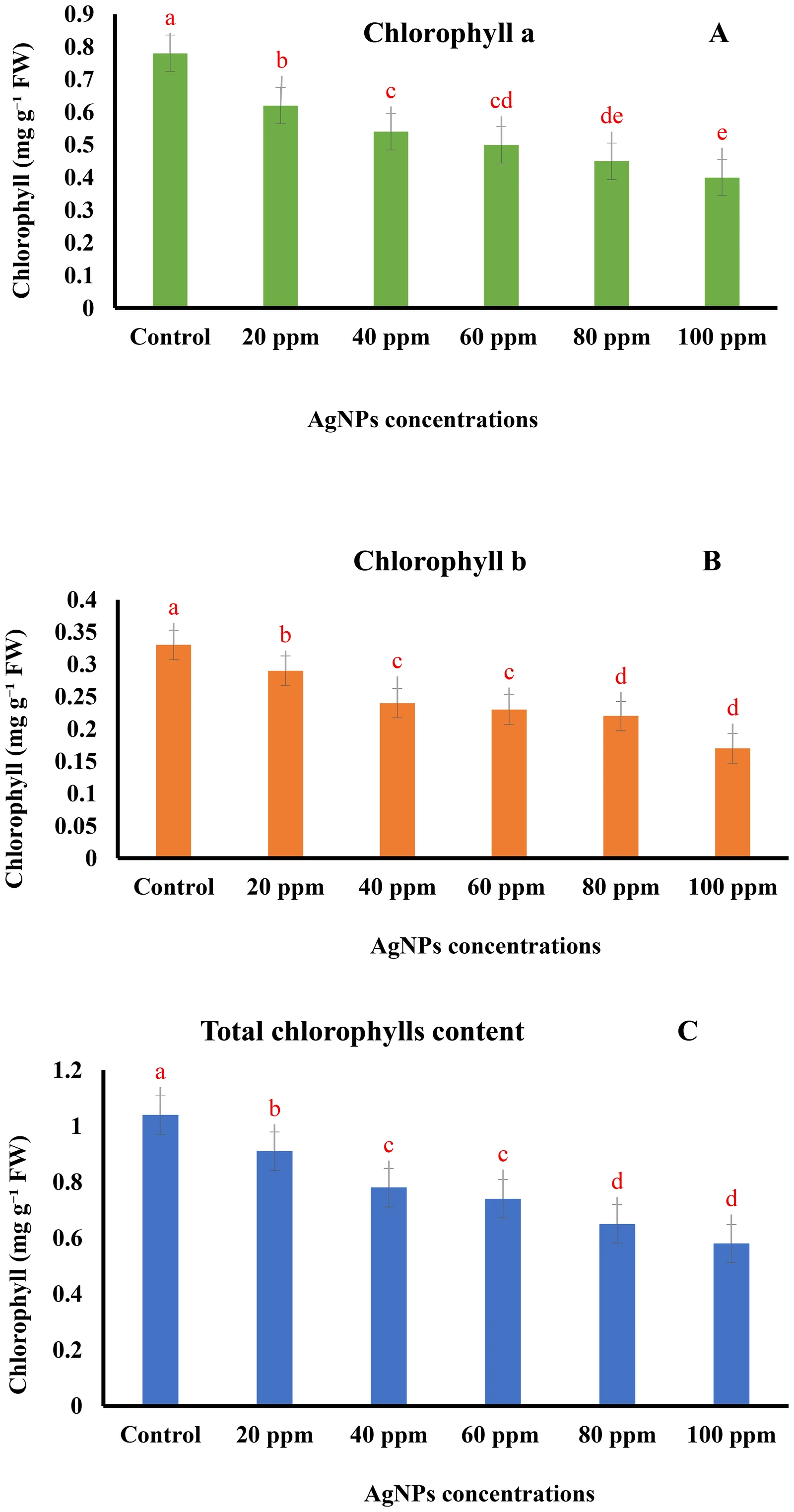
Picture **(A–C)** shown the effects of nanoparticle inoculation on chlorophyll a (Ca), chlorophyll b (Cb), and total chlorophyll contents (mean concentration, mg g^−1^ FW ± SE) in 7-week-old Chinese kale. Bars sharing the same letters are not significantly different at *p*< 0.05 according to Tukey’s Least Significant Difference (LSD) test following one-way ANOVA.

The application of silver nanoparticles (AgNPs) substantially influenced the concentrations of chlorophyll a (Chl a), chlorophyll b (Chl b), and the overall chlorophyll content in 7-week-old Chinese kale (**Figure 3**). The control plants had the most chlorophyll, with Chl a at 0.75 ± 0.004 mg g^−1^, Chl b at 0.33 ± 0.007 mg g^−1^, and total chlorophyll at 1.08 ± 0.003 mg g^−1^. Treatments with AgNPs led to a decrease in chlorophyll content that depended on the concentration. The levels of chlorophyll a (Chl a) dropped from 0.62 ± 0.003 mg g^−1^ at 20 ppm to 0.40 ± 0.143 mg g^−1^ at 100 ppm. Chlorophyll b (Chl b) also went down, from 0.29 ± 0.003 mg g^−1^ at 20 ppm to 0.17 ± 0.004 mg g^−1^ at 100 ppm. The total amount of chlorophyll also went down from 0.91 ± 0.006 mg g^−1^ at 20 ppm to 0.58 ± 0.193 mg g^−1^ at 100 ppm. One-way ANOVA and Tukey’s test (*p*< 0.05) showed that all of the AgNPs concentrations were very different from the control. Also, there were big differences between the different concentrations, except for 40 ppm and 60 ppm, which were not significantly different. These results show that AgNPs lower the amount of chlorophyll in Chinese kale in a way that depends on the dose.

### Effects of AgNPs on Chinese kale fungus pathogens

#### Effect of AgNPs on mycelial inhibition

**Figure 4:**
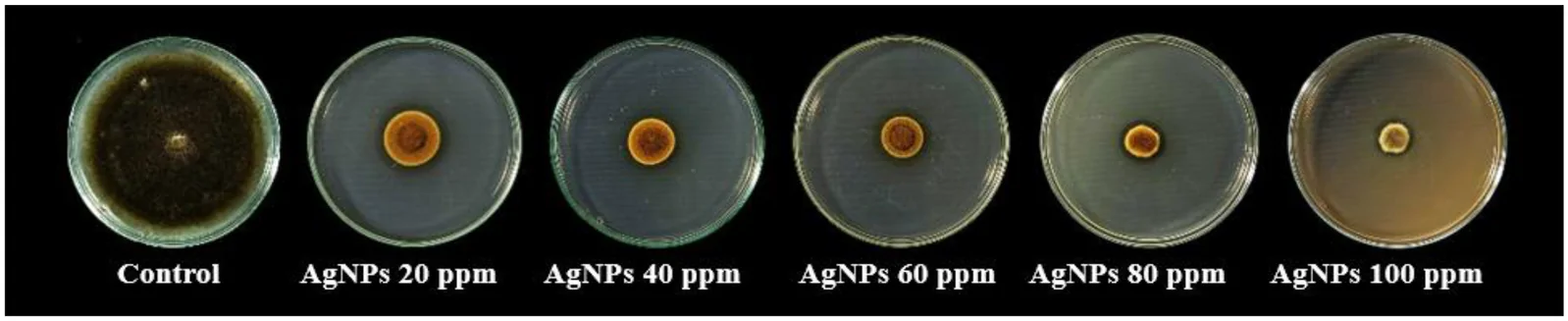
Mycelial inhibition of *A. brassicicola*. After test with AgNPs different concentrations.

The antifungal assay showed that AgNPs significantly slowed the mycelial growth of the *A. brassicicola* isolate CDEP-239 in a way that depended on the concentration (**Figure 4**). As the concentration of AgNPs rose, the inhibition of mycelia increased. At 20, 40, 60, 80, and 100 ppm, the reductions were 68.15%, 75.92%, 77.03%, 83.70%, and 84.44%, respectively (**Table 3**). It is interesting to note that inhibition generally increased with concentration, but at 60 ppm, it was slightly lower than at 40 ppm. This suggests that antifungal activity may not always follow a strictly linear pattern. Still, the highest levels of inhibition were reached at 80 and 100 ppm, which shows that higher amounts of AgNPs significantly inhibited the pathogen's mycelial growth.

**Table 3:** Percentage of mycelial inhibitions of *A. brassicicola* after test with AgNPs difference concentration.

Concentrations							
Treatments	Control	20 ppm	40 ppm	60 ppm	80 ppm	100 ppm	*p-value*
AgNPs	0.00a*	68.15 ± 3.56b	75.92 ± 6.41c	77.03 ± 2.31cd	83.70 ± 0.64cd	84.44 ± 1.11d	<0.001

*a, b, c and d means different letters within the same row indicate a significant difference in mycelial inhibitions caused by the different concentration of the nano particles. ANOVA- Tukey’s Least Significant Difference (LSD) (at *p*< 0.05) was used to determine which concentrations were significantly different.

### Effect of AgNPs on spore germination

The findings indicated that AgNPs significantly inhibited the spore germination of the *A. brassicicola* isolate CDEP-239 in a concentration-dependent manner (**Table 4**, **5**). In the control treatment, conidial germination rose quickly, reaching 62.67% after 3 hours, 89.33% after 6 hours, 94.67% after 9 hours, 95.33% after 12 hours and 100% after 24 hours. On the other hand, all of the AgNPs treatments had strong inhibitory effects. After 24 hours at 20 ppm, spore germination dropped to 19.33%. When the concentration of AgNPs was raised to 40, 60, 80, and 100 ppm, germination dropped even more to 17.67%, 14.33%, 12.33%, and 11.67%, respectively. The inhibition data supported this trend, showing that germination was slowed down by 80.67-88.33% after 24 hours, depending on the concentration. The most effective inhibition was seen at 80 and 100 ppm, where more than 99% of the activity was stopped in the first three to six hours and stayed above 87% even after 24 hours. These results clearly show that AgNPs slow down and stop conidial germination, and that higher concentrations have a stronger and longer-lasting effect.

**Table 4:** Percentage of conidial germination rate of *A. brassicicola* after test with AgNPs difference concentration.

Treatments (ppm)	Germination (%)					
	0 h	3 h	6 h	9 h	12 h	24 h
Control	0.00	62.67 ± 0.88 c*	89.33 ± 3.66 b	94.67 ± 1.33 d	95.33 ± 0.33 d	100 d
AgNPs 20	0.00	2.67 ± 0.33 b	4.33 ± 0.88 a	12.00 ± 0.57 c	15.67 ± 0.66 c	19.33 ± 0.66 c
AgNPs 40	0.00	1.67 ± 0.33 ab	3.67 ± 0.66 a	6.67 ± 0.33 b	14.67 ± 0.66 c	17.67 ± 0.88 c
AgNPs 60	0.00	0.00 a	1.33 ± 0.33 a	3.67 ± 0.33 a	12.00 ± 1.00 b	14.33 ± 0.33 b
AgNPs 80	0.00	0.00 a	0.67 ± 0.33 a	2.67 ± 0.33 a	10.00 ± 0.57 ab	12.33 ± 0.66 ab
AgNPs 100	0.00	0.00 a	0.33 ± 0.33 a	1.33 ± 0.33 a	8.67 ± 0.66 a	11.67 ± 0.33 a
*p*< 0.05	0.00	<0.001	<0.001	<0.001	<0.001	<0.001
*df*	5	5	5	5	5	5
SEM**	0.00	5.59	7.91	8.12	7.54	7.70

*a, b, c and d means different letters within the same column indicate a significant differences in conidial germination rate caused by the different concentration of the nano particles. ANOVA- Tukey’s Least Significant Difference (LSD) (at *p*< 0.05) was used to determine which concentrations were significantly different, **SEM, Standard error of the mean.

**Table 5:** Percentage of conidial inhibition of *A. brassicicola* after test with AgNPs difference concentration.

Treatments (ppm)	Inhibition (%)					
	0 h	3 h	6 h	9 h	12 h	24 h
Control	–	–	–	–	–	–
AgNPs 20	0.00	95.73 ± 0.53 a*	95.13 ± 0.37 a	87.32 ± 0.61 a	83.57 ± 0.7 a	80.67 ± 0.67 a
AgNPs 40	0.00	97.33 ± 0.53 b	95.86 ± 0.37 a	92.96 ± 0.35 b	84.61 ± 0.69 ab	82.33 ± 0.88 a
AgNPs 60	0.00	100 c	98.53 ± 0.37 b	96.12 ± 0.35 c	87.41 ± 1.05 bc	85.67 ± 0.33 b
AgNPs 80	0.00	100 c	99.27 ± 0.37 b	97.18 ± 0.35 cd	89.51 ± 0.61 cd	87.67 ± 0.67 bc
AgNPs 100	0.00	100 c	99.63 ± 0.37 b	98.59 ± 0.35 d	90.91 ± 0.70 d	88.33 ± 0.33 c
*p*< 0.05	0.00	<0.001	<0.001	<0.001	<0.001	<0.001
*df*	4	4	4	4	4	4
SEM**	0.00	0.49	0.51	1.08	0.8	0.83

*a, b, c and d means different letters within the same column indicate a significant differences in conidial inhibition caused by the different concentration of the nano particles. ANOVA- Tukey’s Least Significant Difference (LSD) (at *p*< 0.05) was used to determine which concentrations were significantly different, **SEM, Standard error of the mean.

### Effect of AgNPs on soil chemical properties

Soil chemical properties were evaluated 35 days after treatment using composite rhizosphere soil samples collected from the 100 ppm AgNPs treatment, which showed the highest efficacy against *L. erysimi*, and the control treatment (**Table 6**). The analysis showed differences in several soil chemical parameters between the two treatments. Soil pH was 7.58 in the AgNPs-treated sample and 7.56 in the control sample after the experimental period. Organic matter content was 6.40% in both treatments, whereas total nitrogen was 0.32% in the AgNPs-treated sample and 0.36% in the control. Available phosphorus was lower in the AgNPs-treated sample (543.93 mg kg^-^¹) than in the control (747.75 mg kg^-^¹). Similarly, exchangeable potassium was 323.75 mg kg^-^¹ in the AgNPs-treated sample compared with 740.33 mg kg^-^¹ in the control (**Table 6**). Because these measurements were obtained from composite soil samples without biological replication, the observed differences are presented as descriptive observations and were not subjected to statistical analysis.

**Table 6:** Soil nutrients analysis after treatment of nanoparticles under semi-field condition at 35 days post treatment.

Treatments	Before					After				
	PH	Organic Matter, OM (%)	Total Nitrogen, N (%)	Available Phosphorus, P (mg/kg)	Exchangeable potassium (mg/kg)	PH	Organic Matter, OM (%)	Total Nitrogen, N (%)	Available Phosphorus, P (mg/kg)	Exchangeable potassium (mg/kg)
Soil spray AgNPs 100 ppm	7.15	5.95	0.37	615.88	740.33	7.58	6.40	0.32	543.93	323.75
Control water						7.56	6.40	0.36	747.75	747.75

## Discussions

The current study illustrated that silver nanoparticles (AgNPs) exhibit potent insecticidal and antifungal characteristics while also affecting the physiological attributes of plants and the dynamics of soil nutrients in Chinese kale. These results are consistent with recent developments in nanobiotechnology, which emphasize the dual function of AgNPs as both protectants for plants and regulators of agroecosystem processes (43–46). Insecticidal activities assays exhibited a distinct dose and time dependent impact of AgNPs on *L. erysimi*, with mortality rates significantly escalating at elevated concentrations and extended exposure periods. The decline in LC_50_ values from 43.11 ppm at 24 hours to 19.34 ppm at 48 hours signifies increased susceptibility over time. Similar temporal toxicity patterns have been documented in aphid and lepidopteran pests subjected to metal-based nanoparticles (47–49). The insecticidal action of AgNPs is generally ascribed to the disruption of the insect cuticle, penetration through spiracles, damage to cellular membranes, and the induction of oxidative stress through the generation of reactive oxygen species (ROS) (50, 51). The observed in planta population suppression further substantiates their efficacy in aphid management programs, corroborating prior research indicating that nanoparticle-based formulations may diminish dependence on traditional synthetic insecticides (43, 52). But the clear dose response relationship shows how important it is to optimize concentrations to make sure they work while avoiding any unintended phytotoxic or ecological effects.

Although silver nanoparticles (AgNPs) effectively suppressed aphid populations, higher concentrations adversely affected plant growth parameters. Declines in fresh weight, leaf width, leaf number and chlorophyll content indicate concentration-dependent phytotoxicity. Similar reductions in chlorophyll a, chlorophyll b and total chlorophyll have been reported in leafy vegetables treated with elevated nanoparticle doses (53, 54). These phytotoxic effects are likely associated with oxidative stress, disruption of photosynthetic machinery, and altered nutrient uptake dynamics (55). Nanoparticles can accumulate in leaf tissues, impairing stomatal conductance and chloroplast structure at high exposure levels. Therefore, determining sub-lethal yet effective concentrations is essential to balance pest control with the maintenance of plant physiological integrity.

AgNPs significantly inhibited the mycelial growth and spore germination of *A. brassicicola* in a dose-dependent manner, achieving suppression rates of over 84% to 87% at concentrations of 80–100 ppm. These findings are consistent with numerous studies demonstrating the broad-spectrum antifungal activity of AgNPs against phytopathogens (56, 57). The antifungal mechanism is primarily attributed to the interaction of AgNPs with fungal cell walls and membranes, disruption of membrane permeability, generation of reactive oxygen species (ROS), and inhibition of critical enzymatic pathways (50). The high inhibition rates observed in this study support the integration of AgNPs into disease management strategies, particularly for cruciferous crops susceptible to Alternaria infections.

Soil analysis of the composite rhizosphere samples showed slight differences in pH, organic matter, available phosphorus, and exchangeable potassium between the 100 ppm AgNPs treatment and the control. Because these measurements were obtained from composite samples without biological replication, the observed differences should be interpreted with caution and regarded as preliminary observations rather than statistically validated treatment effects. Previous studies have reported that silver nanoparticles may influence soil chemical properties and microbial-mediated nutrient cycling depending on nanoparticle concentration, soil characteristics, and exposure duration (58, 59). Therefore, further studies incorporating replicated soil sampling and long-term field evaluations are required to determine whether AgNPs application consistently affects soil properties under agricultural conditions.

A limitation of the present study is that batch-specific dispersion stability of the AgNPs working suspensions was not independently characterized by dynamic light scattering, zeta-potential analysis, or time-dependent UV–Visible spectroscopy. Therefore, the suspensions are described as freshly redispersed working suspensions rather than as physicochemically stable colloids. Further studies should evaluate particle-size distribution, surface charge, aggregation behavior, and concentration stability under the actual bioassay conditions.

Overall, this study confirms the potential of silver nanoparticles (AgNPs) as an eco-friendly alternative for managing aphids and fungal pathogens in Chinese kale (60). However, the observed phytotoxic effects and alterations in soil nutrient availability highlight the need for dosage optimization and environmentally responsible application strategies (61). Future research should prioritize long-term field evaluations, the development of controlled release nano-formulations, and comprehensive environmental risk assessments, including impacts on beneficial insects, pollinators, and soil microbiota) (43, 44). Integrating AgNPs within broader integrated pest management (IPM) frameworks may enhance sustainability while minimizing ecological risks (14, 17, 36, 62, 63).

## Conclusions

Silver nanoparticles (AgNPs) exhibited insecticidal activity against *L. erysimi* and antifungal activity against *A. brassicicola* under the experimental conditions of this study. AgNPs reduced aphid populations and inhibited fungal mycelial growth and conidial germination in a concentration dependent manner. Although AgNPs effectively suppressed *L. erysimi* and *A. brassicicola*, higher concentrations adversely affected several plant growth parameters, including fresh weight, leaf width, and chlorophyll content, highlighting the importance of dose optimization. Differences in selected soil chemical properties were also observed following AgNPs application. These findings suggest that AgNPs have potential as a component of integrated pest and disease management in Chinese kale. However, optimization of application rates is essential to achieve effective pest and disease control while minimizing potential adverse effects on plant growth. A limitation of the present study is that TEM, time-dependent DLS, zeta-potential analysis, and analytical verification of the total silver concentration were not performed on the specific AgNPs batch used in the bioassays. Therefore, the reported treatment concentrations represent nominal concentrations, and the previously published characterization data should be interpreted as supporting evidence for the synthesis platform rather than direct batch-specific characterization. Further studies are required to evaluate the long-term effects of AgNPs under field conditions, including their persistence, impacts on soil microorganisms and other non-target organisms, and overall environmental safety before their broader agricultural application.

## Data Availability

The original contributions presented in the study are included in the article/supplementary material. Further inquiries can be directed to the corresponding authors.
